# MOTIVATIONAL INTERVIEWING TO ENGAGE OLDER INPATIENTS IN FALL PREVENTION: PILOT RANDOMIZED CONTROLLED TRIAL

**DOI:** 10.1093/geroni/igz038.1075

**Published:** 2019-11-08

**Authors:** Hiroko Kiyoshi-Teo, Kathlynn Northrup-Snyder, Elizabeth Eckstrom, Debbie Cohen, Nathan Dieckmann, Sydnee Stoyles

**Affiliations:** 1 Oregon Health & Science University, Portland, Oregon, United States; 2 Oregon Health & Science University School of Nursing, Portland, Oregon, United States

## Abstract

Motivational Interviewing (MI) is an evidence-based approach for fostering behavior change and holds potential to engage patients in behavior change related to fall prevention. A two-arm, unblinded, pilot randomized controlled trial was conducted in a hospital setting to determine the feasibility (recruitment and retention), establish suitable procedures for the intervention (duration and quality of MI), and to test study measurements (fear of falling, importance and confidence related to fall prevention, patient activation, fall prevention behaviors, and fall rates). Participants were high fall risk older inpatients (age ≥ 65). The intervention arm received MI at one time during hospitalization in addition to routine hospital fall prevention intervention. The control arm received the routine hospital care for fall prevention only. Measures were collected at baseline, 2-days, 1-week, 1-month, and 3-month. A total of 120 inpatients were contacted by the study team and 67 were randomized: intervention arm (n=36) and control arm (n=31). Approximately 66% of participants completed the study at the 3-month data collection and MI intervention took an average of 21 minutes and was of adequate quality. The intervention group reported less fear of falling after the MI intervention and maintained fall prevention behaviors over time (p<.05). The study identified that MI for fall prevention at a hospital setting was feasible to deliver and provided insights into suitable study procedures and beginning evidence for a positive impact of MI.

